# Exposure to Chemical Cues from Predator-Exposed Conspecifics Increases Reproduction in a Wild Rodent

**DOI:** 10.1038/s41598-018-35568-0

**Published:** 2018-11-21

**Authors:** M. Haapakoski, A. A. Hardenbol, Kevin D. Matson

**Affiliations:** 1 Konnevesi Research Station, Department of Biological and Environmental Science, P.O. Box 35, FI-40014, University of Jyväskylä, Jyväskylä, Finland; 2 Resource Ecology Group, Environmental Sciences Department, Wageningen University, Droevendaalsesteeg 3a, 6708 PB Wageningen, The Netherlands; 3 Present Address: School of Forest Sciences, University of Eastern Finland, P.O. Box 111, FI-80101 Joensuu, Finland

## Abstract

Predation involves more than just predators consuming prey. Indirect effects, such as fear responses caused by predator presence, can have consequences for prey life history. Laboratory experiments have shown that some rodents can recognize fear in conspecifics via alarm pheromones. Individuals exposed to alarm pheromones can exhibit behavioural alterations that are similar to those displayed by predator-exposed individuals. Yet the ecological and evolutionary significance of alarm pheromones in wild mammals remains unclear. We investigated how alarm pheromones affect the behaviour and fitness of wild bank voles (*Myodes glareolus*) in outdoor enclosures. Specifically, we compared the effects of exposure of voles living in a natural environment to a second-hand fear cue, bedding material used by predator-exposed voles. Control animals were exposed to bedding used by voles with no predator experience. We found a ca. 50% increase in litter size in the group exposed to the predator cue. Furthermore, female voles were attracted to and males were repelled by trap-associated bedding that had been used by predator-exposed voles. Movement and foraging were not significantly affected by the treatment. Our results suggest that predation risk can exert population-level effects through alarm pheromones on prey individuals that did not encounter a direct predator cue.

## Introduction

In 1961, Robert Ardrey wrote, “sex is a sideshow in the world of the animal, for the dominant color of that world is fear”^1^. Of course, the purveyors of fear in the world of the animal are predators, and their ultimate threat comes in the form of killing and eating prey. However, predation involves more than just predators consuming prey^2,3^. In fact, when facing only the risk of predation, prey can exhibit an array of anti-predatory responses, which have arisen through co-evolution with predators^4^. Such awareness or perception of predators by prey can affect the physiology, body condition, and reproduction in many prey species (reviewed by Lima 1998^2^). At the population level, the effects of fear of predation may be of the same magnitude or greater than the actual act of predation^5^. While predation fear responses (sometimes placed under the umbrella of “stress”) are often seen as negative or costly for the prey, these responses can be adaptive^6^. For example, fear of predation can result in physiological changes in pregnant females that prepare offspring for life under high predation pressure^7^.

Early predator detection is necessary for prey to maximize their own fitness^8^. In many vertebrate predator-prey systems, the key cue of predator presence is scent^9^. Prey can assess predation risk by eavesdropping on predator scent marks left for intraspecific communication. An adaptive prey response requires recognition and correct interpretation and response to the cue. While fresh cues may suggest predation is imminent and a change in behaviour is needed, old cues should be ignored^10^. Behavioural responses by prey to avoid predation can include reduced activity or escape to another, often lower-quality, habitat^11,12^. These responses can translate to changes in foraging behaviours, decreased body condition^13^, and ultimately reduced fitness. In females, poor body condition can diminish offspring quantity and quality^14^; in males, predator avoidance during the mating season may lead to missed mating opportunities^15^ or breeding delays. Hence, if one looks back at the world described by Andrey, indirect interactions between the sideshow of sex and the world of fear are evident.

Many organisms, including vertebrates, invertebrates, and plants, respond to predation threats by producing alarm signals. (reviewed by Verheggen *et al*.)^16^. For example, some rodents are known to produce alarm pheromones (AP), chemical cues that warn conspecifics about possible dangers^17,18^. Neurobiological and psychological experiments show that individuals can recognise stressed conspecifics from the AP left behind following a stressful situation^19^. The receivers of this indirect signal increase their vigilance and risk assessment behaviour, i.e., change their behaviour in ways that are similar to individuals that encountered a direct predator cue^18^. Furthermore, murine AP is biochemically related to predator-produced scent cues common to most carnivores^20^; both contain similar sulfur-containing volatiles^21^.

In general, a role for fear in ecology of animals is recognized^22^; however, the ecological and evolutionary significance of AP as a fear cue in wild mammals remains unclear. To the best of our knowledge, only two studies have investigated the role of AP in wild mammals. In an experimental indoor arena, Cabrera voles (*Microtus cabrerae*) avoided areas with the scent marks of conspecifics that experienced various predation risks^23^. These predation risks included handling (simulation of capture), audio playbacks of an avian predator, and visual contact with a mammalian predator. In the same study, but under field conditions, Cabrera voles tended to decrease their activity near sites treated with AP. In another study, black-tailed deer (*Odocoileus hemionus columbianus*) produced AP when disturbed or alarmed^24^. In the presence of AP, female black-tailed deer became more alert and left the site more often than in the presence of control odours, odourless air, or deer urine^24^. Most other AP studies have used lab-strain rodents and unnatural stimuli, such as electric foot-shocks^17,18^.

Wild rodents are ideally suited for studying the ecology of predator cues. These animals have a highly developed olfactory system, and odours play a key role in their behavioural decision-making^25^. We tested the hypothesis that wild bank voles (*Myodes glareolus*), a model prey species in many studies of predator-prey interactions, would show antipredator-like responses when exposed to AP^11,26^, since the murine AP shares structural similarity with predator scent^21^. More specifically, we made the following predictions. First, bank voles are expected to decrease movement in response to AP^11^. Second, voles are expected to be more fearful and thus forage less efficiently in response to AP^15,27^. Third, these behavioural and ecological responses should lead to poorer body condition, which in turn should lead to a smaller proportion of breeding females and a smaller litter sizes on average^26,28^.

## Results

**Figure 1:**
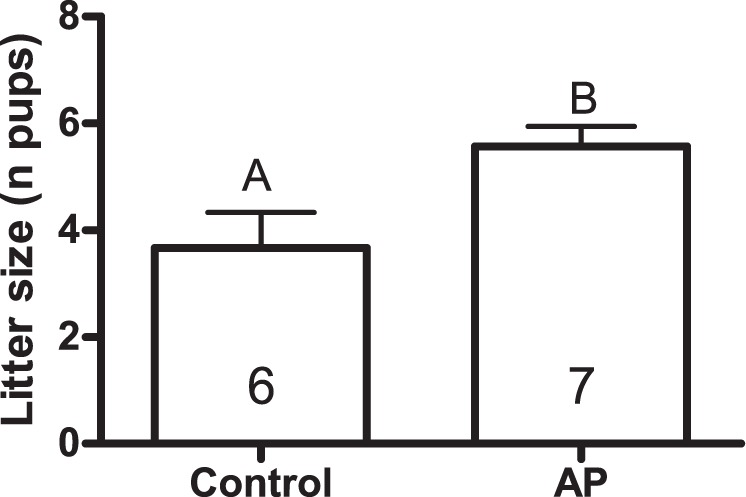
Litter size ± SE in the control treatment, which received bedding materials of non-predator-exposed male bank voles, and in the AP treatment, which received beddings materials of predator- exposed male voles. Sample size inside the bars. Letters denote statistically significant difference between bars.

Litter size was significantly greater by 1.9 ± 0.7 pups in AP enclosures compared to control enclosures (p = 0.013; Table 1, Fig. 1). Previous pregnancy and the interaction between treatment and previous pregnancy were not significant. None of these three terms, which also comprised the pregnancy model, accounted significantly for variation in the proportion of pregnant females (Table 1). Treatment did not have a significant effect on the female survival (control = 55 ± 12%, treatment = 50 ± 10%; Table 1).

**Table 1:** A detailed summary of statistical analysis with the χ^2^ and P values for all fixed factors.

	Litter size			Pregnant females		Female survival	
	d.f.	χ^2^	*P*	χ^2^	*P*	χ^2^	*P*
Treatment*Pregnancy	1	2.31	0.129	1.36	0.243	—	—
Treatment	1	6.23	**0**.**013**	0.55	0.456	0.11	0.741
Pregnancy	1	<0.01	0.980	2.90	0.089	—	—
	**Movement area**			**Trappability**		**Bait-less trapping**	
	**d.f**.	**χ** ^**2**^	***P***	**χ** ^**2**^	***P***	**χ** ^**2**^	***P***
Treatment*Sex	1	0.24	0.627	1.57	0.210	5.46	**0**.**019**
Treatment	1	0.11	0.740	0.87	0.350	—	—
Sex	1	6.34	**0**.**012**	0.42	0.516	—	—
Trappings	1	36.93	<**0**.**001**	x	x	x	x
	**Giving Up Density**						
	**d**.**f**.	**χ** ^**2**^	***P***				
Treatment*Time	1	2.16	0.142				
Treatment	1	0.10	0.749				
Time	1	0.34	0.561				
Vegetation	1	67.51	<**0**.**001**				
No. of Voles	1	0.41	0.520

**Figure 2:**
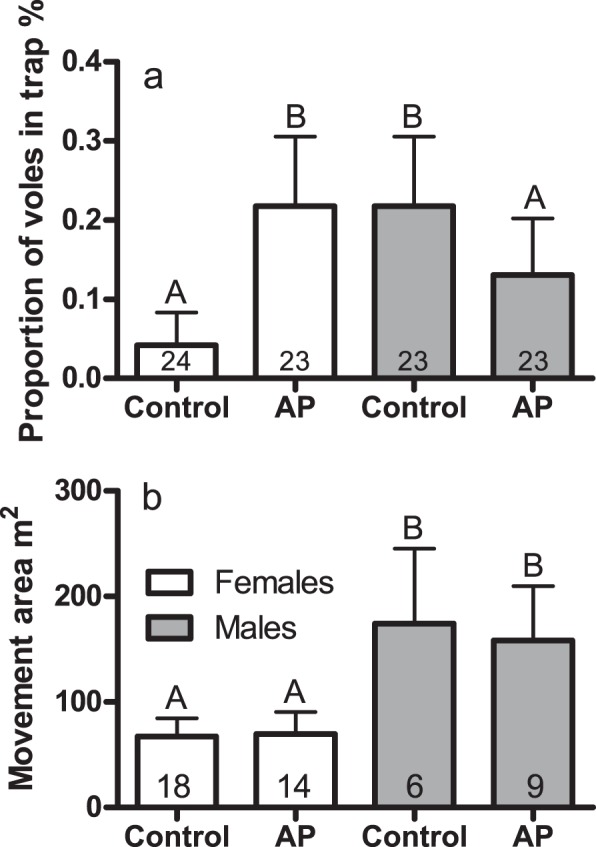
Bait-less trapping proportion ± SE (**a**) and movement area of voles ± SE (**b**) in AP and control enclosures. White bars represent females; grey bars represent males. Sample size inside the bars. Letters denote statistically significant difference between bars.

The interaction between treatment and sex significantly affected bait-less trapping (p = 0.019; Table 1, Fig. 2a). Females were trapped more and males less in the presence of AP. Neither treatment, sex, nor their interaction significantly affected trappability (Table 1). Only sex and the number of trapping instances significantly affected movement: females moved less than males (p = 0.012, females 70 ± 13 m^2^, males 177 ± 45 m^2^; Table 1, Fig. 2b) and more trapping instances correlated with greater movement areas (p < 0.001; 63.48 ± 11.33 m^2^ per trapping instance, Table 1).

**Figure 3:**
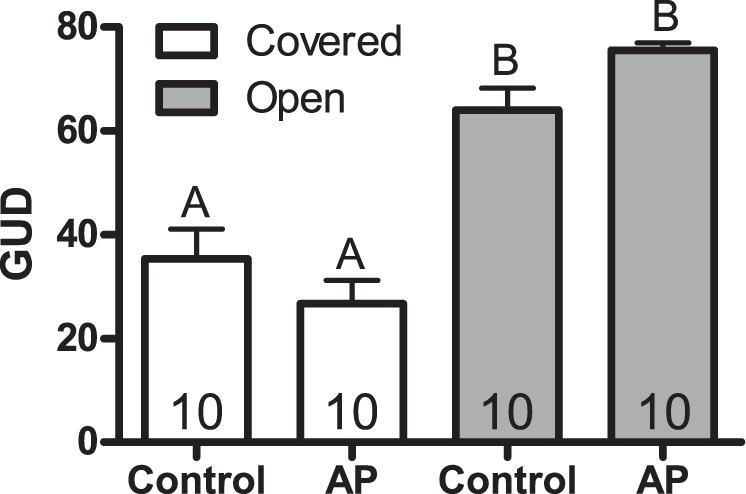
Giving up density (GUD) ± SE measured as number of seed left in the seed-trays in AP and control enclosures. All seed trays contained 80 sunflower seeds mixed with two liters of sand. White bars represent GUD boxes in dense vegetation; grey bars represent GUD boxes in open areas. Sample size inside the bars. Letters denote statistically significant difference between bars.

GUD was significantly higher in areas where the vegetation was open compared to grass-covered areas (by on average 38.8 ± 3.8 seeds, p < 0.001; Table 1, Fig. 3). None of the other model terms accounted significantly for variation in GUD.

## Discussion

### Conspecific alarm cues impact fitness

In the current study, exposure to conspecific AP increased litter size by about 50% compared to control treatment. This result supports our hypothesis that voles can receive different information from predator-exposed and unexposed male conspecifics, and it suggests that female and male voles can respond differently to this information. Chemical cues can serve as an important means of communication among animals, both within and between species. Some rodents AP to warn conspecifics about possible dangers^17,18,23^. AP produced by mice are known to be molecularly similar to chemical cues produced by predators^21^. The observed effects of AP in voles may also be rooted in biochemical similarity, for example, to other compounds that signal danger natural settings, but this possibility requires further investigation.

Increased investment in reproduction by female bank voles faced with a predator cue is in line with several classic hypotheses concerning residual reproductive value^29^ and terminal investment^30^. We conducted the experiment late in the breeding season with the prediction that females should heavily invest resources into current reproduction. Such investment is expected because future reproduction opportunities are improbable for several interacting reasons. The most important are likely the approach of winter and poor overwinter survival of females that have already reproduced^31^, combined with high overall mortality risks from predation^32^. In comparison to AP females, females in the control treatment did not increase their litter size and were still prepared to produce one or two litters during ongoing breeding season. Reproduction should continue as long as there are sufficient resources for doing so; bank vole breeding season in our latitude usually lasts until September^33^. The litter size effect of AP could be mediated by males (e.g., via sperm quality), but examples of such mechanisms are not currently described in the literature. Simple physiological mechanisms could allow bank voles females to increase litter size: bank voles are induced ovulators^34^, meaning that females can produce more pups by mating more with one or several different males^35^. Many animals, including sticklebacks (*Gasterosteus aculeatus*)^36^ and some songbirds, have been found to increase investment in reproduction when predation risk is experimentally increased^37^. However, such increases are not universal. In some cases, cessation or delay of reproduction may result from high levels of predation. This phenomenon has been previously described in bank voles in laboratory studies^38,39^ and in studies with challenging ecological conditions (e.g., variable food availability and predation risk) during the first reproductive window after winter^26^. Reduced reproduction, however, is expected to be an unlikely alternative late in a breeding season. Quantifying fear effect on prey fitness has been criticized because it is difficult to assess the magnitude of experimenter-induced predator cues^40^. While ecological experiments can deviate from natural conditions in some way, we factored in vole biology as much as possible (e.g., by using naturally occurring sex ratios and population densities and by adding scent cues produced by a single unique male per enclosure^41^.

### Males and females respond differently to scent cues

Chemical cues produced by prey faced with a predator might communicate more than just alarm. For example, male lab mice exposed to either a predator (cat urine) or a predator/competitor (rat urine) produce cues that make themselves more attractive to female mice^42,43^. The element of our study involving trapping without seeds as bait offered support for this idea: female voles were more attracted to traps in the AP enclosures, where those females were presented with bedding used by the male voles that had encountered a predator. In contrast, male voles avoided traps in the AP enclosures and were more frequently found in traps in the control enclosures. The fact that only male voles had been used to produce both types of bedding materials in our study may be useful in linking our results to possible mechanisms. One possible candidate attracting females to the bedding materials from predator-exposed AP males could be the major urinary protein (MUP). Mice are able to regulate the total amount of MUP in their urine during social contacts, and female mice advertise their reproductive state by varying the concentration of MUP during the oestrous cycle^44^. MUP can function as a pheromone and stimulate sexual attraction^45^ and oestrus in female mice^46^. The chemical can also promote aggressive behaviour in male mice^47^ and extend the longevity of olfactory cues that they leave behind^48^. One or more urine components (e.g., MUP), and not urine itself, most likely drive our observed differences. Control and AP bedding was collected after being used by a vole for about 48 hours, and since bank voles are assumed to urinate roughly constant rate, both control and AP bedding should have been thoroughly contaminated with urine and feces. Furthermore, prairie voles (*M*. *ochrogaster*) and woodland voles (*M*. *pinetorum*) urine mark at the same rate with and a without predator cue^49^.

If the bedding material used by predator-exposed male voles contained AP that resembled direct odour cues from predators themselves, then we predicted trappability would be higher under control conditions (i.e., unstressed male used bedding), since the bedding materials were distributed in the chimneys near the traps. However, neither the two groups nor the two sexes differed significantly in trappability when baited traps were in use. The absence of an experimental effect might be due to the highly attractive food rewards (i.e., energetically rich sunflower seeds) that were used as a bait in the traps. During the breeding season, voles, especially female ones, have high energy demands, which may be met (at least partially) by consuming the sunflower seed bait. Additionally, the voles in this study may have adjusted to the relatively constant presence of AP, which was present when the voles were added to the enclosure and regularly refreshed until the pregnant females were removed. Ultimately, a behavioural shift favouring the gain of food rewards over the risks of predation may have occurred; such an adjustment matches well with the risk allocation hypothesis^50^.

We also hypothesised that the addition of bedding materials from predator-exposed males would restrict the movement area of voles in the experimental enclosures^51^. Decreasing movement area should effectively decrease the risk of encountering and being killed by a predator^4^. However, we were unable to document an effect of our experimental treatment on movement. Our results contrast with those of an earlier experiment on Cabrera voles, which tended to decrease activity near AP patches^23^. In our enclosures, movement may have been suppressed by the avian predators to such an extent that voles were unable to further reduce their movement in response to our AP treatment^52^. Overall low trappability (around 50%) compared to previous summer experiments (see for example Haapakoski *et al*., 2013^53,54^) supports this finding. Not surprisingly, males were moving more than females regardless of predation risk. Males need to move over several females territory in search of potential mates to increase their fitness^55^.

Perceived predation risk is expected to increase at the expense of foraging^56^. Thus, we expected a higher GUD in enclosures with bedding materials from the predator-exposed voles; however, we were unable to document differences in GUD between treatment and control enclosures. GUD was higher in open areas compared to more densely vegetated areas in our experimental enclosures, suggesting that the GUD method worked. With voles seeming to avoid the avian predation risk associated with foraging in open areas, this result resembles predator facilitation^57^. In fact, previous research suggests that field voles (*M*. *agrestis*) prefer open habitats when facing a mammalian predator (least weasel) and densely vegetated areas when facing an avian predator (kestrel falcon, *Falco tinnunculus*)^52^.

The pregnancy rate was similar in the experimental and control enclosures. Under experimentally increased predation risk in laboratory studies, bank voles have been observed to suppress breeding with females actively avoiding copulation^38^. Yet breeding suppression has never been reported during the peak of the breeding season in the field^58^, and the breeding suppression in the lab might be a laboratory artefact. Breeding suppression or avoidance might only occur when resources are limited^26,59^.

The survival of females did not differ between treatment and control groups. Previous studies of bank voles have not found an effect of predation risk on survival during summer^58,60^, but an inverse relationship between predation risk and survival in winter has been reported^26^. In that case, the assumption was that reduced survival was a consequence of reduced foraging. In our current study, foraging and movement were not significantly affected by the experimental treatment, which might explain the uniformity in survival.

## Conclusions

The effects of predation are multifaceted and extend far beyond the death and consumption of prey. For example, we found that bedding materials used by predator-exposed male voles contained cues AP that could exert strong life-history effects in field settings. Specifically, indirect effects of predation risk, which here was communicated by the presence of the scent cues, translated to 1) increased litter size and 2) an interaction with sex that affected the attractiveness of traps. Thus, AP can impact the fitness and behaviour of animals. Continued study of these cues and others in both field and lab settings will contribute to a more nuanced understanding of interactions between predators and prey and between the sideshow of sex and the world of fear.

## Material and Methods

### Experimental setup

**Figure 4:**
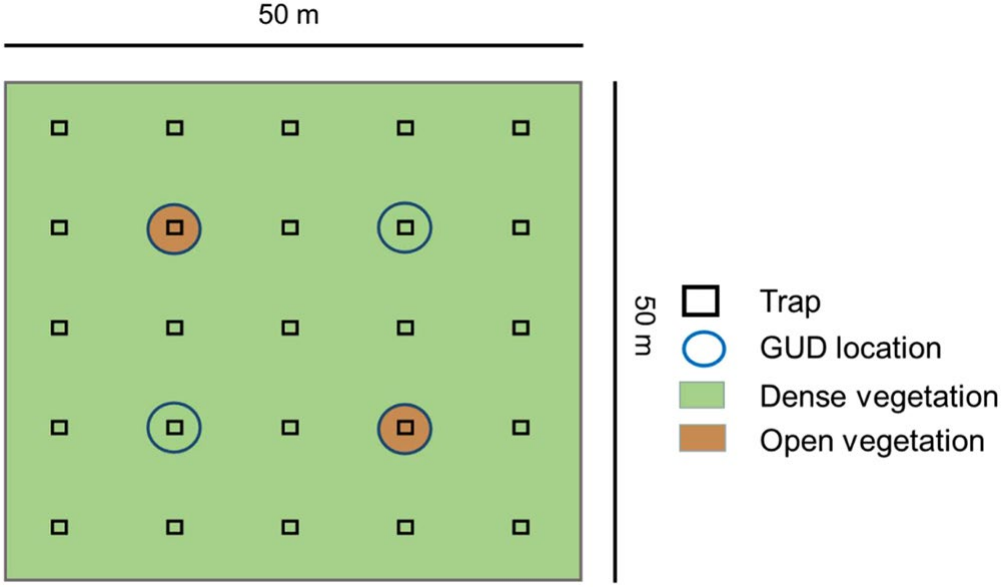
Enclosures used in our experiment were 2500 m^2^, surrounded by a steel wall, and contained 25 traps that had a distance of 10 m between them. At four trapping locations (circles) in each enclosure, we placed GUD boxes, two of which had a non-altered dense vegetation surrounding the boxes and two had an mown open areas surrounding the boxes.

We conducted our experiment in twelve uncovered outdoor enclosures (0.25 ha per enclosure, see Haapakoski *et al*.^26^ for complete details) and in the laboratory of Konnevesi Research Station in central Finland (62°37′N, 26°20′E). Experimental status (AP vs. control, six enclosures each) was randomly assigned. In each enclosure, 25 multiple capture traps (Ugglan Special Nr. 2, Grahnab, Hillerstorp, Sweden) were arranged in a 5 × 5 grid with 10 m intervals between traps (Fig. 4). The traps were covered with bottomless metal chimneys (40 × 40 × 50 cm^3^) on which a metal lid was placed to protect voles in the traps from direct sunlight and rain.

### Study animals

The bank vole (*Myodes glareolus*) is a small common boreal microtine rodent. Vole populations cycle in Scandinavia, and specialist predators play a large role in driving these cycles^61^. One such predator is the least weasel (*Mustela nivalis nivalis*), which specializes in hunting small mammals. Due to its small size, the weasel is able to hunt in the burrows of voles, leaving few safe places for the voles to escape predation^51^. As a consequence of high predation pressure in the wild, antipredator behaviours and physiology are important in bank voles.

In the study area, the breeding season of bank voles usually extends from the end of April to September. During the breeding season, female bank voles are strictly territorial and need an exclusive territory to breed. The territories of breeding males usually overlap with the territories of several females^62^. Bank voles are easy to trap in the field and maintain and breed in laboratory, thereby making the species an ideal subject for the study of predation risk.

We used sexually mature bank voles that were either enclosure- or laboratory-born. These voles were first-generation descendants of wild bank voles that had been originally trapped in the Konnevesi region. Prior to the experiment, these voles were housed individually under standard conditions^60^. Voles grouped together in an outdoor enclosure were unrelated. Enclosure groups were balanced based on vole body size and number of females who had previously given birth.

### Production of alarm pheromone and control bedding materials

To produce AP and control bedding materials, we used six pairs of bank vole brothers from litters produced by six different parental pairs. Each of these twelve male offspring was placed individually into a standard rodent cage containing 3 l of wood shavings. A randomly selected vole from each parental pair was used for AP production, and these six were housed in the same room. The six that produced the control bedding were housed in a separate room.

In order to induce AP production, we exposed the six AP voles to a weasel. For the first two weeks, a caged weasel was placed daily on top of the cage of each AP vole for 2 minutes. Weasel cage was equipped with solid bottom in order to prevent weasel urine and feces mixing in to AP bedding. After this procedure, we captured each AP vole with our hands and released it back into its cage to simulate predator capture. The AP production procedure was changed two weeks after the start of the experiment in order to minimize stress for the weasel. With this new method, a bank vole was first captured by hand from its cage and placed individually inside a small wire mesh cage, which was then placed inside the weasel’s cage for 2 minutes. Afterwards, each vole was released back into its original cage. The weasel used in this procedure was housed under standard conditions^60^ in a cage that was kept in a different building from all voles.

About 48 h old bedding material originating from one specific male vole (either AP or control, see below) was assigned to a specific outdoor enclosure for the duration of the experiment. In all enclosures, 0.1 l of bedding material was placed in each trap chimney and around the giving-up density tray (see below). Bedding material (AP or control) was placed at the start of the experiment and replaced every other day during the experiment.

The experiment started with the spreading AP and control bedding materials into the enclosures at the end of July 2015. On the same day bedding materials were added to an enclosure, four females were released to allow for habituation to the environment and its cue of indirect predation risk or not. Three days after adding the females, four males were released into each enclosure. In order to determine female survival, the proportion of pregnant females, parturition date, and litter size, females were trapped and moved to the laboratory around 18 days after initiation of the experiment.

### Giving-up density

To measure the voles’ perception of predation risk in each enclosure, giving-up density (GUD^56^) was measured using four boxes (20 × 20 × 15 cm^3^ covered with a transparent lids) accessible via two 2 cm diameter entrances. Two GUD boxes were placed in open vegetation, and two were placed under the grass and were thus more protected from avian predators (see Fig. 1). Open vegetation areas were created by mowing the grass in a 1 m diameter area where the GUD boxes were placed. GUD boxes contained 80 sunflower seeds mixed with 2 L of sand. GUD, quantified here the as the number of seeds remaining, was recorded after two consecutive 24 hr periods, which started on the afternoon of day 4 and ending on the afternoon of day 6. If voles perceive that predation risk outweighs foraging gains, then they will give up searching for seeds, and GUD will be high. In situations where they feel safer, the voles will spend more time searching for the diminishing seeds, and GUD will be lower.

### Movement and trappability

To quantify movement area, all traps were activated and regularly checked, and the location of any captured individual was recorded before release at the point of capture. Movement area trapping was conducted on days 10 (evening only), 11 and 12 (both morning and evening), and 13 (morning only). The first movement area trapping (day 10 evening) was conducted without bait (hereafter “bait-less trapping”) to measure the effect of AP treatment, since the food reward might have otherwise lured voles into traps. In bait-less trapping traps were activated immediately after distributing AP and control bedding materials in the chimneys. Bait-less trapping is possible since voles are using trap chimneys and traps as hiding places when traps are deactivated. Movement areas (100% convex polygons) were calculated from the trapping data with the program Ranges VI (Anatrack ltd. Wareham, UK). Movement area calculations excluded individuals that were caught only once. Trappability was calculated per vole as the number of captures divided by six (the number of baited trap checks). Bait-less trapping, used for the calculation of the movement areas but not for trappability. At the start of the intensive trapping period, two individuals were found dead in one trap in one enclosure; these individuals were replaced.

### Statistical analysis

R version 3.2.2^63^ with package lme4^64^ was used to perform linear mixed effects analyses, which accounted for the eight voles per enclosure. We used log-likelihood ratio tests and χ^2^ statistics to evaluate statistical significance (*α* = 0.05). Litter size was analysed by using a model that included treatment (AP/control), previous pregnancy (yes/no) and the interaction between the two. Movement area, trappability and bait-less trapping were analysed by using a model that included treatment (AP/control), sex (male/female), and the interaction between the two. In addition, the number of captures per individual was used as a covariate in the movement area analysis to account for differences in movement area due trappability. Female survival and female pregnancy were analysed with generalized linear mixed models (GLMM) with binomial error distribution. In the analysis of survival, treatment was used as a fixed factor. The pregnancy model, like the litter size model, included as fixed treatment, previous pregnancy (yes/no) and the interaction between the two. GUD was analysed using repeated measures analyses because GUD was measured on two days. For GUD, models included treatment, time (day one/two), their interaction, vegetation (open/covered), and the minimum number of voles known to be alive in the enclosure. Enclosure identity was included as a random factor in all analyses. Residuals were visually inspected for deviations from homoscedasticity and normality. In the movement area analysis, data were cube root transformed to meet the assumptions. For unknown reasons, four voles in one control enclosure died during the movement area trapping. Therefore, we excluded this enclosure from all analyses of data from after the point of mortality. (Data from this enclosure were used in analyses of GUD and bait-less trapping, which were collected before the mortality event.) We also excluded two individuals (IDs 4885 and 4886) in an AP treatment enclosure from our movement analyses, since their tag numbers were indistinguishable in the field.

### Ethics

All work was conducted according Finnish legislation with the animal experimentation permission from Jyväskylä University No. ESAVI/6370/04.10.07/2014.

## Data Accessibility

Data will be deposited to Dryad Digital Repository after acceptance.
